# Monitoring clinical progression with mitochondrial disease biomarkers

**DOI:** 10.1093/brain/awx168

**Published:** 2017-08-03

**Authors:** Hannah E Steele, Rita Horvath, Jon J Lyon, Patrick F Chinnery

**Affiliations:** 1 Wellcome Trust Centre for Mitochondrial Research, Institute of Genetic Medicine, Newcastle University, Newcastle upon Tyne, NE1 3BZ, UK; 2 GlaxoSmithKline, Molecular Safety and Disposition, Ware, SG12 0DP, UK; 3 Department of Clinical Neurosciences, University of Cambridge, Cambridge Biomedical Campus, Cambridge CB2 0QQ, UK; 4 MRC Mitochondrial Biology Unit, Cambridge Biomedical Campus, Cambridge CB2 0QQ, UK

**Keywords:** biomarkers, mitochondrial disease, disease progression, mtDNA, mitochondrial encephalomyopathy

## Abstract

Mitochondrial disorders are genetically determined metabolic diseases due to a biochemical deficiency of the respiratory chain. Given that multi-system involvement and disease progression are common features of mitochondrial disorders they carry substantial morbidity and mortality. Despite this, no disease-modifying treatments exist with clear clinical benefits, and the current best management of mitochondrial disease is supportive. Several therapeutic strategies for mitochondrial disorders are now at a mature preclinical stage. Some are making the transition into early-phase patient trials, but the lack of validated biomarkers of disease progression presents a challenge when developing new therapies for patients. This update discusses current biomarkers of mitochondrial disease progression including metabolomics, circulating serum markers, exercise physiology, and both structural and functional imaging. We discuss the advantages and disadvantages of each approach, and consider emerging techniques with a potential role in trials of new therapies.

## Teaser

The absence of well-characterised biomarkers for mitochondrial disease is restricting the development of new treatments. Steele *et al.* review current and future clinical biomarkers, discuss their relative strengths and weaknesses, and suggest ways forward that will accelerate the clinical translation of new therapies already showing promise in preclinical studies.

## Introduction

Mitochondrial disorders are genetically determined metabolic diseases due to a biochemical deficiency of the respiratory chain that affect around 1 in 4300 of the population in the UK (Gorman *et al.*, 2015). Given that multisystem involvement and disease progression are common features, mitochondrial disorders carry substantial morbidity and are associated with excess premature death (Kaufmann *et al.*, 2011). Despite this burden, a recently published Cochrane review did not identify any disease-modifying treatments of benefit (Pfeffer *et al.*, 2012), and current best management of mitochondrial disease is therefore supportive. Consequently there is an unmet need for treatments that modify the underlying biochemical deficit and disease trajectory.

However, the development of new therapeutic strategies presents major challenges for the scientific, pharmaceutical, academic and clinical communities (Food and Drug Administration, 2004). For rare diseases, including mitochondrial disorders, these issues are magnified through the geographical dispersion of patients, the use of heterogeneous patient groups in both interventional and natural history studies to date, and historically at least, a perceived lack of return on financial investment for the pharmaceutical industry (Pfeffer *et al.*, 2013). As biomarker identification provides a means of overcoming some of these barriers—for example, enabling prospective compounds to be assessed in a practical timescale, or facilitating patient subgroup categorization—their development has been afforded high scientific priority (Food and Drug Administration, 2004).

**Figure 1:**
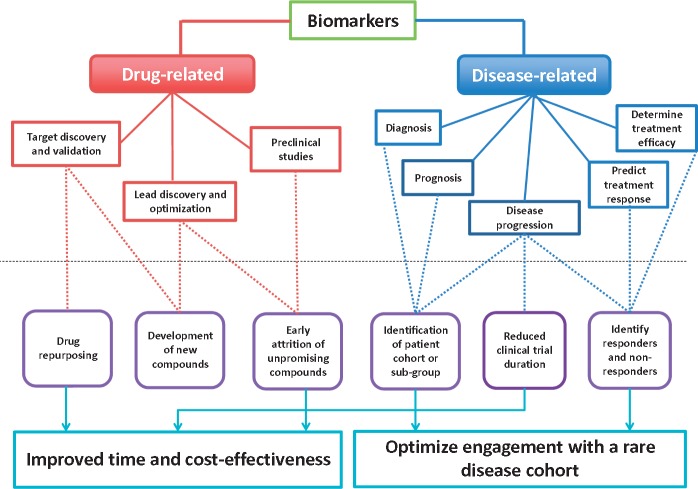
**Potential impact of biomarkers on clinical trials**.

Biomarkers are widely defined as: ‘A characteristic that is objectively measured and evaluated as an indicator of normal biologic processes, pathogenic processes, or pharmacologic response(s) to a therapeutic intervention’ (Biomarkers Definitions Working Group, 2001). With increasing scientific focus on developing biomarkers, an expansive nomenclature has emerged (Biomarkers Definitions Working Group, 2001; Altar *et al.*, 2008; Wagner, 2008). ‘Drug-related’ biomarkers are those identifying novel pathways, or enabling assessment of drug-target interactions; while ‘disease-related’ biomarkers reflect the presence or absence of disease, aid disease stratification, guide prognosis or inform disease natural history (Fig. 1). Following identification of a potential biomarker, it should be both validated and qualified: where validation refers to the process of determining assay reliability, and qualification describes the process of linking a biomarker with biological processes and clinical endpoints. Where biomarkers have a specific purpose, in a specific patient cohort, their impact on clinical trial design is outlined in Fig. 1.

Traditionally, biomarkers in mitochondrial diseases have been used to improve diagnostic accuracy or target those who should undergo invasive investigation. However, as next generation sequencing techniques progressively improve the diagnostic process (Taylor *et al.*, 2014), alternative uses of biomarkers can be increasingly explored. As several therapeutic strategies for mitochondrial disorders are now at a mature preclinical stage (Bogacka *et al.*, 2005; Lagouge *et al.*, 2006; Yatsuga and Suomalainen, 2012), and are making the transition into early-phase patient trials (Table 1), it is the lack of validated biomarkers for disease progression that currently presents the biggest challenge in developing new therapies for patients.

**Table 1:** Active clinical trials of potential disease modifying agents for primary mitochondrial diseases, ClinicalTrials.gov April 2017

Study title	Phase	Design	IMP
The effect of arginine and citrulline supplementation on endothelial dysfunction in mitochondrial diseases	II	R, PC, DB, CO	Arginine, citrulline
Study to assess the efficacy and safety of Raxone in LHON patients (LEROS)	IV	OL	Idebenone
An exploratory, double-blind, randomized, placebo-controlled, single-center, two-way cross-over study with KH176 in patients with the mitochondrial DNA tRNALeu(UUR) m.3243A>G mutation and clinical signs of mitochondrial disease	II	R, PC, DB, CO	KH173
A study of bezafibrate in mitochondrial myopathy	II	OL	Bezafibrate
RTA 408 capsules in patients with mitochondrial myopathy - MOTOR	II	R, PC, DB	RTA408
Efficacy study of GS010 for the treatment of vision loss up to 6 months from onset in LHON due to the ND4 mutation (RESCUE)	III	R, Sham C, DB	GS010
EPI-743 for metabolism or mitochondrial disorders	II	R, PC, DB, CO	EPI-743
MNGIE allogeneic hematopoietic stem cell transplant safety study (MASS)	I	OL	Hematopoietic allogeneic stem cells
A study investigating the safety, tolerability, and efficacy of elamipretide (MTP-131) topical ophthalmic solution for the treatment of Leber’s hereditary optic neuropathy	II	R, PC, DB	MTP-131
Safety study of an adeno-associated virus vector for gene therapy of Leber’s hereditary optic neuropathy (LHON) caused by the G11778A mutation (LHON GTT)	I	OL	scAAV2-P1ND4v2
Long term safety and efficacy study of EPI-743 in children with Leigh syndrome	II	R, PC, DB	EPI-743

CO = crossover; DB = double blinded; IMP = investigational medicinal product; OL = open label; PC = placebo controlled; R = randomized; Sham C = sham controlled.

The unmet need for surrogate markers of disease progression becomes clear when considering three key clinical features of mitochondrial diseases. First, the disorders are notoriously heterogeneous—even within genetically homogeneous groups. Second, acute relapses are frequently experienced; and third, baseline progression tends to occur slowly over a number of years. In the context of clinical trials, and particularly early phase trials that tend to be conducted over short timescales (e.g. 6 weeks to 6 months), these factors present significant barriers in determining therapeutic efficacy. Given this, our review will focus on scientific approaches to identify biomarkers of clinical disease progression in mitochondrial disorders with a unique emphasis on emerging preclinical techniques. We are aware that mitochondrial abnormalities may be secondary to various cellular processes including calcium metabolism, neurode generation and various metabolic diseases; however, the role of mitochondria in these diseases needs further investigations (Pyle *et al.*, 2015). Therefore, we focus this update on primary mitochondrial disorders.

## Metabolomics

A fundamental feature of primary mitochondrial disorders is deficient oxidative phosphorylation, with both up- and downstream metabolic perturbations arising secondarily. Abnormalities in lactate, pyruvate, creatine kinase, amino acids and carnitines are firmly established in the clinical investigation of mitochondrial diseases. However, their diagnostic sensitivity and specificity is poor (Petty *et al.*, 1986; Campos *et al.*, 1993; Jackson *et al.*, 1995; Sim *et al.*, 2002; Jeppesen *et al.*, 2006*a*; Haas *et al.*, 2008; Suomalainen *et al.*, 2011; Yamada *et al.*, 2012; Davis *et al.*, 2013) and there are limited natural history studies studying change in relation to disease progression. To date, metabolomic approaches have been highly successful in identifying potential biomarkers in a diverse range of disorders including: cancers (Puchades-Carrasco *et al.*, 2013; Rocha *et al.*, 2015; Cui *et al.*, 2016), vascular disease (Jove *et al.*, 2015; Naz *et al.*, 2015), renal transplantation (Kienana *et al.*, 2015), respiratory diseases (Adamko *et al.*, 2015), immunological disorders (Young *et al.*, 2013; Saegusa *et al.*, 2014), liver disease (Gao *et al.*, 2015), and diabetes (Balderas *et al.*, 2013; Drogan *et al.*, 2015). With secondary mitochondrial dysfunction arising in several of these conditions (Wallace, 2012; Yu *et al.*, 2012; Begriche *et al.*, 2013; Cloonan and Choi, 2016), alongside the association of diabetes with primary mitochondrial diseases, the application of metabolomics in genetically determined mitochondrial cohorts is timely and holds great potential.

For a multi-systemic disorder, a particularly attractive feature of metabolomic analysis is that it can be undertaken using CSF, blood, urine, saliva, or solid biopsy samples. It is unknown at present which of these is optimal in mitochondrial disorders specifically, but non-invasive (urine/saliva) or minimally invasive (blood) samples would facilitate repeated measurements in both longitudinal and therapeutic settings. Initial study of the urinary proteome and metabolome in patients with heterogeneous mitochondrial disorders identified key differences between carriers, healthy controls and those manifesting symptoms (Hall *et al.*, 2015).

Analysis can be undertaken using either nuclear magnetic resonance spectrometry (NMR spectrometry) or mass spectrometry (MS) techniques. NMR spectrometry can utilize samples in both solid and liquid states; is highly reproducible, and is better at analyte quantification than mass spectrometry (Emwas, 2015). However, mass spectrometry techniques have significantly higher sensitivity than NMR spectrometry, enabling detection of analytes at low concentrations (femto–attomolar) thereby facilitating recognition of metabolites not traditionally measured in routine clinical practice, with a relevant example being the alteration of sphingomyelins and phosphatidylcholines in a cohort with Leber’s hereditary optic neuropathy (Chao de la Barca *et al.*, 2016).

Furthermore, data arising from metabolomics studies can be used in a variety of clinically meaningful ways. In addition to genotype or phenotype specific cohort analysis identifying specific disease manifesting and carrier ‘signatures’ (Hall *et al.*, 2015), longitudinal ‘n-of-1’ studies can be undertaken to facilitate analysis of metabolomic change with an individual’s clinical disease progression (Alonso *et al.*, 2015). The ability to combine these approaches is particularly attractive and could help with the presymptomatic identification of ‘disease onset’ in carrier individuals—a factor of clear importance for clinical trial design.

## Circulating serum markers

### Circulating cytokines

In the past 5 years, serum fibroblast growth factor-21 (FGF-21) and serum growth and differentiation factor-15 (GDF-15) (Kajiyama *et al.*, 1989; Suomalainen *et al.*, 2011; Davis *et al.*, 2013; Kalko *et al.*, 2014; Fujita *et al.*, 2015; Yatsuga *et al.*, 2015) have emerged as two promising diagnostic biomarkers for mitochondrial diseases. Identified first in mouse models of mitochondrial disease they were subsequently validated in patient cohorts. Presently, both markers are more sensitive and specific than currently used clinical diagnostic markers of mitochondrial disorders but are yet to be incorporated into formal diagnostic pathways (Suomalainen *et al.*, 2011; Yatsuga *et al.*, 2015; Davis *et al.*, 2016). In part, this has been due to concerns that both have been associated with a range of non-mitochondrial disorders, encompassing obesity, cancer, renal disease, diabetes, and liver disease, with the latter two frequently co-existing in patients with mitochondrial disorders (Semba *et al.*, 2012; Chow *et al.*, 2013).

Although preliminary data suggested that FGF-21 may correlate with disease severity and disease progression (Suomalainen *et al.*, 2011), this was not subsequently substantiated in adult cohorts with the m.3243A>G mutation (Koene *et al.*, 2014, 2015). Their utility in determining disease progression and severity therefore needs further assessment in broader, well characterized mitochondrial cohorts. Additionally, it is not known whether they are influenced by therapeutic agents, but both markers should be considered for inclusion as study endpoints in relevant clinical trials, particularly as emerging work suggests that GDF-15, and FGF-21 in particular, appear to be more specific markers for mitochondrial disorders arising due to mitochondrial translation and mtDNA maintenance defects, as opposed to those resulting from impaired respiratory chain complex or assembly factors (Lehtonen *et al.*, 2016).

### MicroRNAs

MicroRNAs are non-coding genomic regions, around 20 nucleotides long, that control gene expression through transcription silencing. Several studies have used serum microRNAs in the diagnosis of inherited muscle disease (Cacchiarelli *et al.*, 2011; Endo *et al.*, 2013; Hu *et al.*, 2014), and serum circulating, muscle-specific microRNAs have been linked to disease progression in myotonic dystrophy (Koutsoulidou *et al.*, 2015). Distinctive microRNA patterns have also been associated with various metabolic processes including non-alcoholic fatty liver disease (Leti *et al.*, 2015), diabetes (Raffort *et al.*, 2015), brown adipogenesis (Zhang *et al.*, 2015), as well as exercise capacity in healthy individuals (Mooren *et al.*, 2014). Their interaction with the mitochondrial genome has not been fully elucidated but a recent study in cybrid cells carrying the m.3243A>G mutation identified that microRNA-9/9* patterns associated with mitochondrial disorder phenotypes [mitochondrial encephalomyopathy, lactic acidosis and stroke-like episodes (MELAS) and myoclonic epilepsy with ragged-red fibres (MERRF)] (Meseguer *et al.*, 2015). Given that serum samples are straightforward to collect, further study of microRNAs in relation to mitochondrial disease phenotype and disease progression would be rapidly feasible.

## Exercise physiology

The functional assessment of mitochondrial aerobic capacity in the adult patient population has been extensively studied using exercise physiology for over 10 years. Key differences are seen in peak oxygen consumption (peak VO_2_), peak power (W_max_), and peak arterial-venous oxygen difference between individuals with mitochondrial disorders and healthy controls (Jeppesen *et al.*, 2003, 2006*b*; Bates *et al.*, 2013). As an indicator of oxygen uptake from the capillary network during circulation, the reduced peak arterial-venous oxygen difference seen in those with mitochondrial disorders is believed to be a key mechanism underpinning the widespread experience of exercise intolerance (Jeppesen *et al.*, 2006*b*; Bates *et al.*, 2013).

To date, exercise testing has been used both to support a diagnosis of mitochondrial disease (Jensen *et al.*, 2002) and to demonstrate efficacy of treatment (Drinkard *et al.*, 2010; Glover *et al.*, 2010), including when exercise is used as a therapy itself (Taivassalo *et al.*, 1998, 2001, 2006; Jeppesen *et al.*, 2006*a*, *b*, 2009; Murphy *et al.*, 2008; Bates *et al.*, 2013). To our knowledge, no longitudinal studies using exercise physiology parameters as markers of disease progression have been undertaken in a mitochondrial cohort to date. While exercise testing is safe in mitochondrial populations, there are several potential limitations with its use in this way. First, studies have largely focused on those with myopathic symptoms and application to other mitochondrial phenotypes is likely to require further exploration. Second, little is published on the exercise capacity of children with mitochondrial disorders, although a small study reviewing exercise as a therapeutic intervention did not identify problems with the exercise itself (Schreuder *et al.*, 2010). Third, participants with cardiac involvement and significant intellectual or physical disabilities would be unable to exercise at the level required for a valid test. Finally, the test requires specialist equipment and trained staff to administer which could limit its widespread use and reliability in longitudinal settings.

In addition to maximal exercise testing, recent work on gait physiology has identified distinct abnormalities in the m.3243A > G and m.8344A > G mitochondrial populations (Galna *et al.*, 2014). These characteristics correlate with disease severity and furthermore, affected individuals can be distinguished from healthy controls at an early disease stage.

## Imaging

Imaging findings are being increasingly identified in a broad range of neuromuscular disorders, including as markers of disease progression (Morrow *et al.*, 2013, 2016). The main focus is on the measurement of muscle volume, and the relative amount of intramuscular fat and water (reviewed in Carlier *et al.*, 2016). The majority of studies to date have been carried out in patients with inherited muscular dystrophies and inflammatory myopathies, but similar features have also been shown in mitochondrial diseases, suggesting potential applications for the diagnosis and monitoring of these disorders. However, large longitudinal studies have not been performed to date. The array of imaging available to study the impact of mitochondrial disorders encompasses structural imaging using CT or magnetic resonance, to functional imaging using magnetic resonance spectroscopy (MRS) and PET. All modalities are established in diagnostic and research settings and provide non-invasive, quantitative measurements in a variety of tissues, making it attractive to patients and researchers alike, particularly as stronger magnetic fields (7 T) and novel software algorithms enable shorter scan times.

### Structural imaging

Structural brain imaging using MRI is well described in mitochondrial disorders. While common clinical features include cerebral and cerebellar atrophy, bilateral high signal in deep grey structures, leukoencephalopathy and stroke-like episodes in non-vascular territories (reviewed in Saneto *et al.*, 2008); these findings are non-specific and highly variable (Tzoulis *et al.*, 2006; Engelsen *et al.*, 2008), making them unsuitable for further development as biomarkers. In contrast, extra-ocular muscle T_2_ signal in individuals with chronic progressive external ophthalmoplegia (CPEO) correlates with eye movement restriction thereby providing a quantitative method of assessing disease severity (Yu-Wai-Man *et al.*, 2013; Pitceathly *et al.*, 2016). Similarly, in a cohort with the m.3243A > G mutation, structural cardiac abnormalities have been identified in the absence of both high symptom load and cardiac abnormality on routine clinical tests (Hollingsworth *et al.*, 2012). Furthermore, the specific pattern of cardiac involvement appears dependent on patient genotype and is distinct from more common causes of cardiac impairment (Florian *et al.*, 2015). Longitudinal studies using these modalities would be relatively straightforward, and both would be potentially attractive as trial endpoints in appropriate cohorts.

### Functional imaging with existing techniques

#### Magnetic resonance spectroscopy

MRS enables the quantitative assessment of tissue metabolites in steady state, with ^31^P and ^1^H spectra most commonly used in mitochondrial cohorts. ^1^H enables capture of tissue-specific metabolites such as lactate, choline, and *N*-acetyl aspartate, while ^31^P captures the relative proportions of phosphorus metabolites. Although skeletal muscle can be examined by ^31^P-MRS in resting, exertional or post-exertional states, the evaluation of oxidative capacity depends on measuring the phosphocreatine recovery time—a direct reflection of mitochondrial ATP production—following phosphocreatine depletion through exercise.

^31^P-MRS on skeletal muscle has identified key differences in tissue bioenergetics in a range of neuromuscular disorders, including mitochondrial disease (Lodi *et al.*, 1999, 2004*b*; Cea *et al.*, 2002; Jeppesen *et al.*, 2007). To date these metabolic alterations have been used to support a diagnosis of mitochondrial disease (Walker *et al.*, 1996; Bernier *et al.*, 2002) and as an endpoint in several therapeutic studies (Penn *et al.*, 1992; Barbiroli *et al.*, 1995, 1997; Kornblum *et al.*, 2005). However, some involved single patients only (Penn *et al.*, 1992; Barbiroli *et al.*, 1995) while in others, trial design was not optimal (Pfeffer *et al.*, 2012). Similar abnormalities of tissue bioenergetics have also been identified using ^31^P-MRS of cardiac muscle in those carrying the m.3243A > G mutation (Lodi *et al.*, 2004*a*), and have been used to assess the effectiveness of an exercise intervention programme (Bates *et al.*, 2013). Longitudinal studies correlating clinical disease progression in mitochondrial cohorts with findings from ^31^P-MRS studies are notably lacking.

While impaired ATP production and elevated lactate levels are established findings of brain MRS studies (Barbiroli *et al.*, 1993; Kaufmann *et al.*, 2004; Lindroos *et al.*, 2009), little has been published on the use of brain MRS to monitor disease progression or to study the effects of pharmaceutical agents (Barbiroli *et al.*, 1999; Lee *et al.*, 2010). Recent work using ^1^H-MRS brain imaging has identified fundamental differences between healthy controls, those manifesting disease due to the m.3243A>G mutation, presymptomatic individuals converting to affected individuals and presymptomatic individuals not converting (Weiduschat *et al.*, 2014); once more supporting the notion of specific disease state ‘signatures’ (Hall *et al.*, 2015). Natural history studies to further understand this in relation to both symptom progression and in broader mitochondrial patient populations would be timely and highly relevant given current interest in biomarker development.

#### Positron emission tomography

In contrast to MRS, which provides steady state measurement of metabolites, PET measures metabolic flux, thereby permitting the study of tissue metabolic and haemodynamic properties. Several radioisotopically labelled metabolites are relevant to the study of mitochondrial disorders and include ^15^O, 2-deoxy-2 ^18^F-fluoro-d-glucose (FDG) and ^11^C pyruvate enabling the study of tissue-specific bioenergetics. To date, PET has identified a variety of metabolic abnormalities in the mitochondrial disease population. MELAS patients with the m.3243A>G point mutation are the best studied population, with reports of PET imaging of heart (Arakawa *et al.*, 2010), brain (Nariai *et al.*, 2001; Shelly *et al.*, 2007; Lindroos *et al.*, 2009) and muscle tissue (Arakawa *et al.*, 2010; Rodan *et al.*, 2015), both in background states (Lindroos *et al.*, 2009), acute post stroke-like episodes (Shelly *et al.*, 2007; Ikawa *et al.*, 2009), and in response to potential therapies (Arakawa *et al.*, 2010). Key findings from cerebral studies include: global impairment of cerebral oxygen metabolic rate (CMRO_2_) (Nariai *et al.*, 2001; Lindroos *et al.*, 2009) and regional glucose hypometabolism of the occipito-parietal regions (Nariai *et al.*, 2001; Shelly *et al.*, 2007). These perturbations are supported by findings from heterogeneous mitochondrial populations, which include reduced molar ratio (of glucose and oxygen) (Frackowiak *et al.*, 1988); impairment of CMRO_2_ (Frackowiak *et al.*, 1988; Shishido *et al.*, 1996); and reduced cerebral metabolic ratio glucose (CMRglu) (Frackowiak *et al.*, 1988; Haginoya *et al.*, 2016), including in a family with mitochondrial neurogastrointestinal encephalopathy (MNGIE) with no clinically overt CNS features (Lehnhardt *et al.*, 2008). Additionally, perturbations in cerebral oxygen extraction fraction (OEF) have been identified, with the OEF representing the percentage of oxygen removed from the blood by tissue during the passage through the capillary network (Frackowiak *et al.*, 1988; Lindroos *et al.*, 2009). It is therefore analogous to the ‘arterial-venous oxygen difference’ measured during sub-maximal exercise testing, although no studies exist that examine the relationship between the two. Further study could therefore be warranted although PET scanning has several limitations impeding its widespread use in both clinical and research settings. In particular, due to the use of radioactive isotopes (Duncan *et al.*, 1995), there are no natural history studies of mitochondrial populations, and restricted studies in children with mitochondrial disorders.

#### Functional MRI

These limitations have provided impetus to develop magnetic resonance protocols that facilitate study of tissue haemodynamics. MRI using gradient echo sampling of spin echo (GESSE) sequences has emerged as a technique enabling quantitative assessments of cerebral haemodynamics, in particular the OEF (He and Yablonskiy, 2007). Key advantages of OEF measurement over cerebral blood flow, are its relative uniformity despite regional variations in cerebral blood flow or oxygen metabolic rate (Gusnard *et al.*, 2001) and its interindividual stability (He and Yablonskiy, 2007). Initial application in a small MELAS (m.3243A>G) cohort demonstrated reduced cerebral OEF irrespective of relationship to stroke-like episode, with further reduction in OEF in the acute and subacute phases of the stroke-like episode (Yu *et al.*, 2013).

Similar techniques to enable measurement of skeletal muscle OEF have already been applied in small cohorts of healthy individuals (Zheng *et al.*, 2014; Wang *et al.*, 2016) and future assessment in patients with mitochondrial disorders would be relevant given the reduction in tissue oxygen extraction (peak arterial-venous oxygen difference) widely identified during sub-maximal exercise tests in these cohorts (Taivassalo *et al.*, 2006). Should the muscle OEF technique be further assessed and validated, it could have advantage over submaximal exercise testing in a mitochondrial cohort because of its application to those unable to exercise, for example, those with significant weakness, cardiovascular disease, children or intellectual disabilities.

### Functional imaging with novel techniques

With conventional FDG-PET and NMR spectroscopy both lacking the necessary sensitivity to identify substrates in low tissue concentrations, researchers have developed a relatively new MRI technique—dynamic nuclear polarization (DNP). DNP facilitates real time functional imaging, using ^13^C-MRS, of substrates and their metabolites in existence at low tissue concentrations—such as reactive oxygen species—*in vivo* (Ardenkjaer-Larsen *et al.*, 2003). Although hyperpolarized [1-^13^C] pyruvate has been the most widely studied substrate, enabling both functional imaging of key tumour metabolites (Brindle *et al.*, 2011; Nelson *et al.*, 2013) and cellular response to chemotherapy (Day *et al.*, 2007; Ward *et al.*, 2010), use of hyperpolarized [1-^13^C] glucose permits more direct study of the glycolytic pathway.

To date, a key limitation in the technique has been the short half-life of the hyperpolarization period (10–40 s for pyruvate; <1 s for glucose), necessitating the ensuing metabolic processes to occur rapidly, and resulting image generation to occur in <2 min (Brindle *et al.*, 2011). Deuteration is one means of overcoming this challenge and recently, hyperpolarized, deuterated [U-^2^H, U-^13^C] glucose was used to image glycolysis in real time (Rodrigues *et al.*, 2014). Initial results suggest the technique allows the sensitive study of lactate accumulation in murine cancer models pre- and post-chemotherapy.

Such techniques, although relatively early in development, have clear application in mitochondrial disease, potentially providing a minimally invasive, yet quantitative, means of diagnosis—as well as a way of monitoring the systemic, or indeed tissue-specific, impact of a given treatment. DNP is under active development in cancer medicine and requires further validation in man before preliminary assessments are made in mitochondrial disease specifically.

Preclinical work is also underway using novel PET ligands, for example 1^8^F-BCPP-EF, to enable quantitative analysis of complex I activity. To date the technique has been used following induced ischaemia in monkey brain (Tsukada, 2014; Tsukada *et al.*, 2014) and its application to primary dysfunction of mitochondria has not been established. However, the use of ligands with the ability to interrogate specific mitochondrial complexes may in future enable the non-invasive assessment of respiratory chain function.

## Emerging techniques

### Small molecule reporters

Small molecule reporters enable the quantifiable measurement of mitochondrial function, mitochondrial-specific metabolites and reactive oxygen species generation *in vivo.* Tailor-made probes, administered intravenously to an intact organism, accumulate within mitochondria and react with a substrate of interest. In doing so, the probes are modified to produce an ‘exomarker’ (exogenous marker), which can then be extracted to enable its quantitative analysis, and inferences to be made about the reacting substrate. Currently, the technique is in preclinical development, with analysis of the exomarker using mass spectrometry only being feasible following destruction of the organism (Logan *et al.*, 2014). However, it is anticipated that further study of exomarkers in urine may facilitate analysis of probes *in vivo* and facilitate future work in animal models and eventually, humans.

Although current focus of this technique is in the assessment of acquired mitochondrial dysfunction arising due to ischaemic and reperfusion insults (Chouchani *et al.*, 2014), the potential application to primary respiratory chain disorders is evident. Such techniques would provide an attractive way of ensuring drug delivery to mitochondria and quantifying drug activity (Porteous *et al.*, 2010; Hoogewijs *et al.*, 2016); as well as hypothetically determining optimal drug dosing for individual patients, or facilitating the direct assessment of mitochondrial function in relation to disease progression.

### Cutaneous respirometry

The ability to objectively measure respiratory chain function *in vivo*, non-invasively, and without need for imaging, has the potential to revolutionize the follow-up and treatment of those with mitochondrial disorders. Cutaneous respirometry has been developed by a Dutch research team investigating mitochondrial dysfunction arising due to sepsis (Harms *et al.*, 2015). The device, which sits over the sternum, can measure both mitochondrial oxygen tension (mitoPO_2_) and oxygen consumption (mitoVO_2_). It does this by using the oxygen dependent optical properties of protoporphyrin-IX, a haem precursor synthesized within mitochondria, known as the PpIX-triplet state lifetime technique. Testing in healthy volunteers is at an early stage, but has confirmed that mitoPO_2_ and mitoVO_2_ measurements are viable (Harms *et al.*, 2016). Furthermore, aside from minor local skin reactions, the device was well tolerated. The application to mitochondrial patients is evident and would be particularly suitable, following further assessment of reliability and validity, in the setting of a natural history study, clinical trial or even routine clinic appointment.

## Discussion

While mitochondrial disorders unite on a final common metabolic pathway, their heterogeneous, multi-systemic and fluctuating nature provides particular challenges in the identification of biomarkers correlating with overall disease progression. Functional imaging studies and exercise physiology are well established for the diagnosis of mitochondrial disorders, and both converge on impaired tissue oxygen extraction. However, their role in measuring disease progression is less clear, and much could be gained through well designed longitudinal studies of genotyped cohorts using these modalities. The combination of bio-energetic and structural imaging is particularly promising, particularly in evaluating mitochondrial cardiomyopathy (Ng *et al.*, 2016), but, the need for specialist equipment and personnel conceivably limits their use. In contrast, circulating serum markers, such as FGF-21 and GDF-15; microRNA analysis and metabolomic assessment are appealing because the samples are relatively easy to collect in both paediatric and adult mitochondrial populations. However, it is currently not clear whether any serum or blood-based biomarkers will be useful as endpoints in clinical efficacy trials, particularly in groups that are genetically and clinically heterogeneous, or where a tissue-specific phenotype of interest falls within a multi-system mitochondrial disease syndrome. Longitudinal studies in patient cohorts are required to clarify whether these approaches will have a role in the future, or whether the integration of different datasets using multivariate statistical methods, for example, may help identify collections of markers that together better reflect the complex nature of disease progression. Furthermore, as ‘signatures’ reflecting carrier status have already been identified (Weiduschat *et al.*, 2014; Hall *et al.*, 2015), the field needs to consider whether inclusion of asymptomatic carrier individuals in observational and/or interventional studies should be facilitated.

Patients and their families want the least burdensome and clear means to monitor progression of their disease so that clinicians can provide timely and appropriate support. Clinicians and industry partners urgently need new biomarkers to facilitate clinical development of promising novel treatments. How best to reach these goals and capitalize on the advances in biomarker techniques discussed is a critical issue that needs to be collectively addressed by clinicians, scientists, industry partners and patients, to ensure a strategic, integrated and acceptable approach is taken.

## Funding

P.F.C. is a Wellcome Trust Senior Fellow in Clinical Science (101876/Z/13/Z), and a UK NIHR Senior Investigator, who receives support from the Medical Research Council Mitochondrial Biology Unit (MC_UP_1501/2), the Wellcome Trust Centre for Mitochondrial Research (096919Z/11/Z), the Medical Research Council (UK) Centre for Translational Muscle Disease (G0601943), EU FP7 TIRCON, and the National Institute for Health Research (NIHR) Biomedical Research Centre based at Cambridge University Hospitals NHS Foundation Trust and the University of Cambridge. The views expressed are those of the author(s) and not necessarily those of the NHS, the NIHR or the Department of Health. R.H. is a Wellcome Trust Investigator (109915/Z/15/Z), who receives support from the Medical Research Council (UK) (MR/N025431/1), the European Research Council (309548), the Wellcome Trust Pathfinder Scheme (201064/Z/16/Z) and the Newton Fund (UK/Turkey, MR/N027302/1). GlaxoSmithKline provides support for H.E.S. as part of their Early Talent Review Board (ETRB) Clinical Fellow program.

## Abbreviations

MELAS: mitochondrial encephalomyopathy, lactic acidosis and stroke-like episodes
MRS: magnetic resonance spectroscopy
NMR: nuclear magnetic resonance
OEF: oxygen extraction fraction
